# Atomic Scale Origin of Metal Ion Release from Hip Implant Taper Junctions

**DOI:** 10.1002/advs.201903008

**Published:** 2020-01-21

**Authors:** Shanoob Balachandran, Zita Zachariah, Alfons Fischer, David Mayweg, Markus A. Wimmer, Dierk Raabe, Michael Herbig

**Affiliations:** ^1^ Department Microstructure Physics and Alloy Design Max‐Planck‐Institut für Eisenforschung GmbH Max‐Planck‐Straße 1 Düsseldorf 40237 Germany; ^2^ Department of Orthopedic Surgery Rush University Medical Center 1611 W. Harrison St. Chicago IL 60612 USA

**Keywords:** biomedical titanium alloys, cobalt–chromium–molybdenum alloys, Morse taper junctions, total hip replacement, tribocorrosion

## Abstract

Millions worldwide suffer from arthritis of the hips, and total hip replacement is a clinically successful treatment for end‐stage arthritis patients. Typical hip implants incorporate a cobalt alloy (Co–Cr–Mo) femoral head fixed on a titanium alloy (Ti‐6Al‐4V) femoral stem via a Morse taper junction. However, fretting and corrosion at this junction can cause release of wear particles and metal ions from the metallic implant, leading to local and systemic toxicity in patients. This study is a multiscale structural‐chemical investigation, ranging from the micrometer down to the atomic scale, of the underlying mechanisms leading to metal ion release from such taper junctions. Correlative transmission electron microscopy and atom probe tomography reveals microstructural and compositional alterations in the subsurface of the titanium alloy subjected to in vitro gross‐slip fretting against the cobalt alloy. Even though the cobalt alloy is comparatively more wear‐resistant, changes in the titanium alloy promote tribocorrosion and subsequent degradation of the cobalt alloy. These observations regarding the concurrent occurrence of electrochemical and tribological phenomena are vital to further improve the design and performance of taper junctions in similar environments.

## Graphical abstract

Corrosion and wear at taper junctions in metallic implants can release toxic metal ions into the body. Although biomedical cobalt alloys have a higher hardness than titanium alloys, this study finds that changes on the titanium alloy during fretting can promote tribocorrosion in the cobalt alloy. Such insights can be used to improve material design in taper junctions and implants.

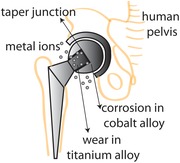

## 1 Introduction

Arthritis, an informal term encompassing a wide variety of joint pains or joint diseases, is a leading cause of disability worldwide. In the United States alone, 52.5 million adults were affected by arthritis in 2010 and 78.4 million adults will be affected by 2040.^1^ Hip and knee osteoarthritis has been ranked as the 11th highest cause of global disability.^2^ For patients with chronic pain or severely limited mobility, Total Hip Arthroplasty (THA) is widely regarded as a highly successful operation improving their quality of life.^3^, ^4^, ^5^, ^6^ During THA, the diseased hip is replaced with a ball‐and‐socket joint designed to mimic the biomechanics of the hip. The ball‐and‐socket assembly can consist of a metallic head articulating either on a metallic cup (metal‐on‐metal implants: MoM) or on a polyethylene lining (metal‐on‐polymer implants: MoP). The global demand for THAs has been predicted to rise;^7^, ^8^ a study of 20 countries projects that hip surgeries will increase from 1.8 million in 2015 to 2.8 million in 2050.^9^ Failure of an implant necessitates revision surgery, which is more expensive and carries higher risk of complications for the patient than the primary THA.^10^, ^11^ Hence, the reliability of hip implant materials is a growing concern since THAs are increasingly being performed on younger patients with more active lifestyles and longer life expectancies.^12^

In order to ease surgery as well as facilitate revision without removing a well‐fixed stem from the femoral bone,^13^ modularity, or the separation of various components of the implant, was introduced. Hence in modern designs, the “ball” in the ball‐and‐socket joint is made of a cobalt alloy (Co–Cr–Mo) femoral head and titanium alloy (Ti‐6Al‐4V) femoral stem coupled via a Morse taper junction^14^ to utilize the superior wear resistance of cobalt alloy articulating surfaces^15^ as well as osseointegration properties^16^ and lower elastic modulus^17^ of the titanium alloy. This head‐stem interface is susceptible to both fretting (micro‐motion) due to cyclic loading as well as corrosion due to the ingress of biological fluids. The ensuing wear debris and corrosion products released from the taper junction accumulate in the hip joint cavity, possibly leading to Adverse Local Tissue Reactions (ALTRs),^18^ pseudotumors,^19^, ^20^, ^21^ and/or osteolysis (degeneration of bone). The latter may cause aseptic loosening and fracture of the implant.^22^ Metal ions released from the implant may be transported into the bloodstream,^23^, ^24^ causing systemic effects. Cobalt has been associated with thyroid, cardiac, and neurological dysfunction^25^ while Cr^6+^ ions are carcinogenic.^26^

Cases of ALTRs due to fretting cobalt–titanium alloy taper junctions have been reported in MoM^27^, ^28^ as well as MoP implants.^29^, ^30^ Alerts on MoM hip implants have been issued by the U.S. Food and Drug Administration due to concerns about metallic debris from the articulating cobalt alloy bearing surfaces (i.e., between the ball and socket).^31^ However, cobalt–titanium alloy taper junctions in MoP implants have also been documented to release corrosion products and cause ALTRs^32^, ^33^, ^34^ similar to the MoM‐bearing surfaces. A recent clinical study concluded that cases of ALTRs were significantly higher in patients carrying a modular MoM implant (46%) compared with a non‐modular MoM (16%) and that fretting corrosion at modular junctions in cobalt‐titanium alloy couples was clinically more significant than wear of the MoM bearing.^35^

Cobalt and titanium alloys are known to be highly corrosion‐resistant due to the formation of passive oxide layers on the surface.^36^ The oxide film on the cobalt alloy has a higher hardness and fracture strength compared to the oxide layer on the titanium alloy.^37^, ^38^, ^39^ In spite of this, significant transfer of cobalt and chromium to the titanium alloy^40^ and higher rates of degradation in the cobalt alloy compared to the titanium alloy^41^ were observed in cases of in vivo fretting. The mechanisms leading to these effects are still being debated. During in vitro studies of fretting corrosion in cobalt–titanium alloy couples, the mechanical (variation of inter asperity distance with load) and electrochemical (fretting current) measurements^38^ as well as overall corrosion damage^5^ were observed to depend on the cobalt alloy rather than the titanium alloy, again for reasons that are unknown. The relative corrosion resistances of the cobalt–titanium alloy couple observed in vitro have indicated that no galvanic corrosion takes place.^42^ The abrasive wear of the cobalt alloy head by the titanium alloy stem has been suggested to occur due to in vivo oxidation of the titanium alloy, which increases its surface hardness above that of the cobalt alloy.^43^ On the other hand, Fischer et al. concluded that the fretting behavior is dominated by delaminations on the titanium alloy resulting in microploughing and subsequent corrosion in the cobalt alloy.^44^

To summarize, the wear and corrosion mechanisms as well as associated ion release pathways occurring during fretting of cobalt–titanium alloy couples in taper junctions are to date unclear. Recognizing that changes in the subsurface play a role during fretting, we investigate here the nano‐scale structure and near‐atomic scale 3D elemental distribution of the subsurfaces of a cobalt‐titanium alloy couple that was subjected to in‐vitro gross‐slip fretting in bovine calf serum with 3 mm H_2_O_2_. This scenario reflects loading conditions of hip joint taper junctions in an inflammatory periprosthetic environment. For this purpose, we employ the correlative transmission electron microscopy (TEM) and atom probe tomography (APT) method, which is a novel high resolution technique to investigate nano‐ and atomic‐scale phenomena involving both structural and chemical features.^45^, ^46^ We provide evidence that a folding mechanism takes place in the subsurface of the titanium alloy that leads to the formation of raised shelves on the surface. These raised shelves formed on the titanium alloy microplough the cobalt alloy surface and hence promote tribocorrosion in the cobalt alloy, thus leading to the release of metal ions into the surrounding serum. During the folding process, surface oxides (from both the titanium and cobalt alloy), the interfacial medium, as well as cobalt and chromium (from the corroding cobalt alloy) are incorporated into the subsurface of the titanium alloy.

## 2 Results

### 2.1 Titanium and Cobalt Alloy Systems

**Figure 1:**
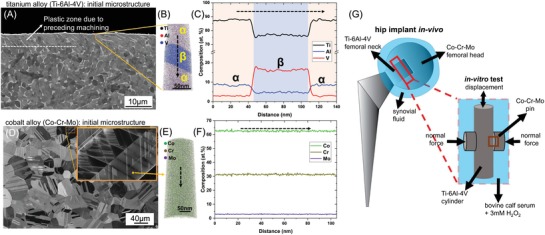
Initial microstructure and composition of the titanium (Ti‐6Al‐4V) and cobalt (Co–Cr–Mo) alloys before fretting. A) SEM‐BSE image of the cross‐section of the titanium alloy showing the α (area fraction ≈ 90%) and β phases. B) APT reconstruction showing the distribution of elements in the subsurface. C) 1D composition profile along the dashed arrow in (B), with the composition of the α and β phases indicated. D) ECCI image of the cobalt alloy surface. E) APT reconstruction of the cobalt alloy subsurface. F) 1D composition profile along the dashed arrow in (E), showing the homogenous composition. G) Schematic representation of the in vivo fretting location in a modular hip implant and the in vitro fretting test.

The microstructural analysis of the titanium and cobalt alloys (before the fretting tests) is shown in **Figure** **1**. The initial microstructure of the titanium alloy (Ti‐6Al‐4V, DIN EN ISO 5832–3) contains both α (hcp, ≈90% area fraction) and β (bcc) phases in an α + β phase field processed condition (Figure 1a). The α phase has a “globularised” morphology due to recrystallization under thermomechanical processing.^47^ The edge of the cylinder has a plastic zone (≈5 µm depth) with elongated α due to preceding sample machining. APT reconstruction of the subsurface (in the plastic zone) provides a quantitative 3D atom map of the distribution of the constituent elements and elemental partitioning between the α and β phases (Figure 1b). The composition of titanium, aluminum, and vanadium in the α phase are 87.5, 9, and 3 at%, respectively, and that in the β phase are 77.5, 5, and 16.5 at%, respectively (Figure 1c). Such elemental partitioning is typical for Ti‐6Al‐4V alloys processed in the α + β temperature regime.^48^, ^49^

Electron channeling contrast imaging (ECCI) of the mechanically polished surface of the cobalt alloy (Co‐Cr‐Mo, DIN EN ISO 5832‐12) prior to fretting reveals the typical microstructure of a wrought processed Co–Cr–Mo alloy without carbides (Figure 1d).^50^ Equiaxed grains with twins and crystalline defects such as stacking faults and partial dislocations are visible (Figure 1e inset). Electron backscatter diffraction (EBSD) analysis confirms that the matrix is γ‐fcc (not shown). Figure 1f is the APT reconstruction of the subsurface of the cobalt alloy. The APT reconstruction and the 1D‐concentration profile extracted from it (Figure 1f) reveals that the cobalt alloy has a homogenous composition of ≈63 at% Co, ≈30.5 at% Cr, and ≈3 at% Mo. Si (1.7 at%) and Mn (0.6 at%) are present as well, which are within the allowed standard limits for this material.

To elucidate the mechanisms of fretting corrosion, a curved cobalt alloy pin was fretted against a titanium alloy cylinder in a medium consisting of bovine calf serum and 3 mm H_2_O_2_, as schematically illustrated in Figure 1g. The normal load, frequency, and amplitude of displacement were chosen to mimic the average human gait cycle in the gross slip regime (detailed in the Experimental section). A multiscale characterization was conducted, including inductively coupled plasma‐optical emission spectroscopy, scanning, and transmission electron microscopy and correlative methods of atom probe tomography, to break down the structural and chemical evolution of the fretted area from a microscopic to a near atomic length scale. After 50 000 fretting cycles, both the surfaces were cleaned with enzymatic soap to remove the organic fraction of the tribomaterial formed between the surfaces. The measured gravimetric wear loss in both the titanium and cobalt alloy after the removal of the tribomaterial was within the range of resolution of the scale (0.1 mg), suggesting that the mass loss was very small under the chosen parameters.

### 2.2 Titanium Alloy after Fretting

#### 2.2.1 Wear Surface of the Titanium Alloy

**Figure 2:**
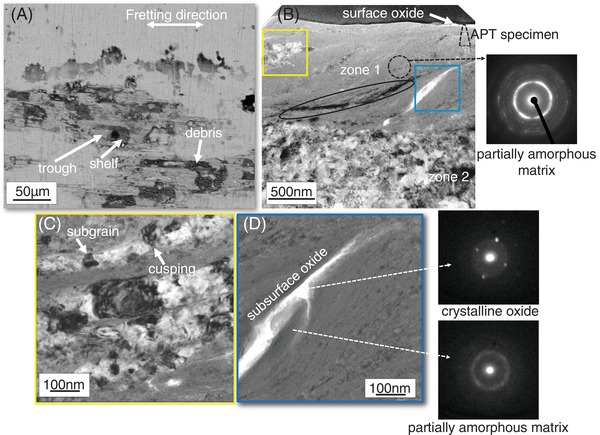
Microstructure of the wear surface of the titanium alloy after fretting. A) SEM‐BSE image of the prominent features on the wear surface: raised shelves and troughs filled with grainy debris. B) TEM‐BF image of the cross‐section of a shelf. Oxides are present on the surface and the subsurface consists of zone 1 (refined matrix phase) and zone 2 (ultrafine equiaxed grains). The diffraction pattern from zone 1 (dotted circle) indicates a partially amorphous matrix. C) TEM‐BF image of cusping and subgrain formation inside the α ribbons seen in the yellow square in B). D) STEM‐BF image of the subsurface oxide seen in the blue square in (B). Nanobeam diffraction patterns indicate that the oxide is crystalline and the surrounding matrix is partially amorphous.

The microstructure of the titanium alloy after fretting is shown in **Figure** **2**. The prominent features on the wear surface of the titanium alloy (Figure 2a) are raised shelves, troughs adjacent to the shelves and grainy debris filling the troughs.

#### 2.2.2 Subsurface of the Titanium Alloy

The TEM bright field image (BF) of the cross‐section of a shelf is given in Figure 2b. An oxide layer is present on the surface (marked in Figure 2b). The subsurface microstructure consists of two zones: a near‐surface zone (zone 1 in Figure 2b) containing a refined matrix phase, beneath which is a zone of ultrafine equiaxed grains (zone 2 in Figure 2b). In the near‐surface zone 1, ribbon‐like features of different thicknesses are laminated along with the refined matrix phase. Selected area diffraction indicates that the matrix region (dotted circle in Figure 2b) is partially amorphous. Inside ribbon‐like features corresponding to the α phase (yellow square in Figure 2b), cusping, and subgrain formation has occurred (Figure 2c). Comparatively thinner ribbons, appearing as long dark bands (ellipse in Figure 2b), contain similarly oriented subgrains of the α phase. The rest of the ribbons (blue square in Figure 2b) were found to be oxides (Figure 2d). We infer that these are surface oxides incorporated into zone 1 during the fretting test. Nanobeam diffraction (NBD) scans (spatial resolution of ≈2 nm) show that the oxide is crystalline while the surrounding matrix is partially amorphous (Figure 2d).

#### 2.2.3 Chemical Composition of the Subsurface of the Titanium Alloy

**Figure 3:**
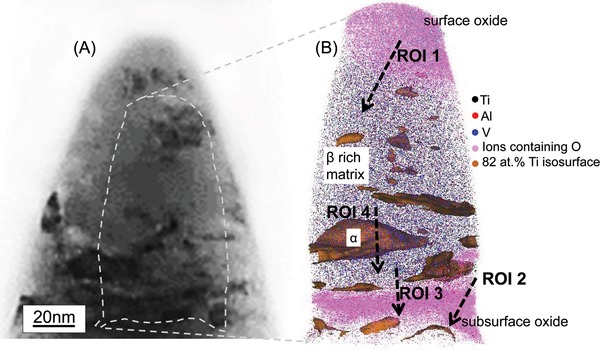
Subsurface of a shelf found on the titanium alloy wear surface after fretting. A) STEM‐HAADF image of the APT specimen from the shelf subsurface. B) corresponding APT reconstruction of the shelf surface showing the surface and subsurface oxides, fragmented α particles and the diffuse interface between the subsurface oxide and β matrix. The approximate position of the APT specimen is marked in Figure 2B. The fragmented particles are isosurfaces generated from a defined range of concentrations. Due to the slight composition variation between the fragments, some features of the STEM image (Figure 3a) are not highlighted in the APT reconstruction (Figure 3b). The regions of interest (ROI 1 to 4) marked by dashed arrows are locations of the 1D composition profiles that will be depicted in Figure 4.

**Figure 4:**
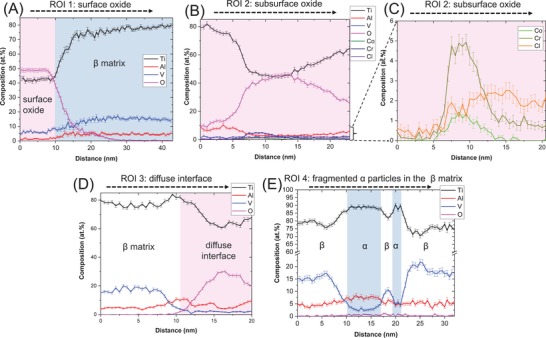
1D composition profiles of the subsurface features in the titanium alloy after fretting. The profiles are extracted from the regions of interest marked in the APT reconstruction in Figure 3B. A) ROI 1: surface oxide and its interface with the matrix, B) ROI 2: subsurface oxide band. C) ROI 2: Magnified view of (B) showing the constituents of the tribomaterial and cobalt alloy present in the subsurface oxide band. D) ROI 3: diffuse interface, between the subsurface oxide band and the matrix, that is enriched in oxygen. E) Titanium rich fragmented α particles in the matrix.

For further investigating the laminate features in zone 1, an APT specimen was lifted from this zone (marked in Figure 2b) and correlative microscopy was performed on it (**Figure** **3**). The STEM‐high angle annular dark field (STEM‐HAADF) image of the APT tip in Figure 3a provides an overview of the phase and composition distribution based on combined atomic and diffraction contrast. The APT reconstruction (Figure 3b) of the area highlighted in Figure 3a shows the distribution of titanium, aluminum, vanadium, and oxygen containing ions in the β‐rich matrix as well as iso‐surfaces of near α phase compositions of titanium (≈82 at%). Oxygen rich regions (pink regions in Figure 3b) correspond to surface or subsurface oxides. The 1D composition profiles across various regions of interest (ROI 1–4), marked by dashed arrows in the APT reconstruction (Figure 3b), are given in **Figure** **4**.

The surface oxide (ROI 1), which shares a boundary with the β‐rich titanium matrix, is compositionally near to TiO (with the presence of aluminum and vanadium), as seen in Figure 4a. Mass/charge spectrum peaks corresponding to TiO_2_ were also detected. The interface between the surface oxide and the titanium (β) matrix is defined by a sharp (≈5 nm thick) boundary.

The composition profile across a subsurface oxide (ROI 2) is given in Figure 4b. The varying oxygen concentration indicates that the boundary of the subsurface oxide with the matrix is relatively diffuse, unlike the surface oxide‐matrix interface in Figure 4a. The composition of the central region of the subsurface band is similar to the surface oxide, supporting our hypothesis that lamination of the surface oxide into the titanium alloy subsurface (seen in Figure 2b,d) occurred. In addition, the subsurface oxide region also contains chromium (≈5 at%), cobalt (≈1 at%), and chlorine (≈2 at%), as shown in Figure 4c. The chromium/cobalt composition ratio indicates that the chromium in the subsurface oxide band must originate from the chromium oxide rich layer on the surface of the cobalt alloy rather than from the bulk of the cobalt alloy. (The chromium/cobalt composition ratio in the bulk of the cobalt alloy is ≈0.4). The diffuse interface between the subsurface oxide band and the β phase matrix is imaged across a different location of the subsurface oxide band in ROI 3. As seen in the corresponding 1D composition profile in Figure 4d, there exists an intermixing zone enriched in oxygen bordering the matrix region (which has negligible oxygen). The oxygen content in this zone is lower than that of the subsurface oxide shown in Figure 4b. This intermixing zone between the subsurface oxide and the matrix corresponds to the partially amorphous matrix surrounding the subsurface oxide identified in Figure 2d.

Titanium‐rich fragmented particles (82 at% Ti isosurfaces) are distributed inside the β‐rich matrix phase (Figure 3b). Figure 4e is the composition profile across one such titanium rich fragmented particle and the β rich matrix phase (ROI 4). The α and β phases can be distinguished by their different compositions and an additional smaller fragmented α particle (≈2.5 nm in width) is detected. The subgrain orientation in the α ribbon‐like features observed in Figure 2b,c indicates that such fragmented particles of α could form by shearing along slip bands arising from planar slip.^51^

### 2.3 Cobalt Alloy after Fretting

#### 2.3.1 Wear Surface of the Cobalt Alloy

**Figure 5:**
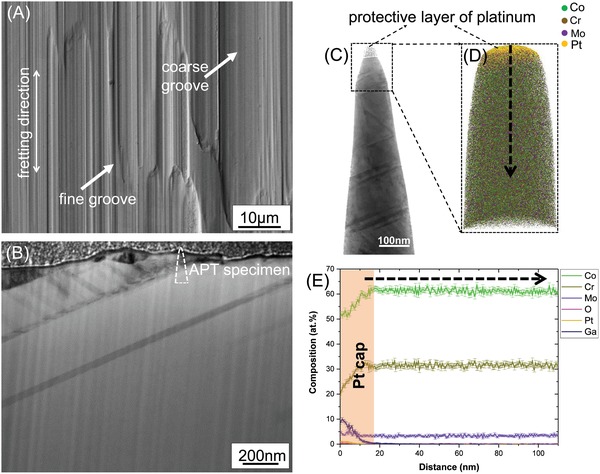
Microstructure of the wear surface of the cobalt alloy after fretting. A) SEM‐BSE image of the prominent features on the wear surface of the cobalt alloy: fine and coarse grooves. B) STEM‐BF image of the subsurface of a groove. C) STEM‐BF image of the APT specimen and D) corresponding APT reconstruction of the cobalt alloy subsurface. E) 1D composition profile along the dashed arrow shown in (D).

Since the constituent elements of the cobalt alloy appear inside the subsurface of the titanium alloy (Figure 4c), it is of interest to investigate the changes on the cobalt alloy wear surface and subsurface after the fretting tests and removal of the tribomaterial (**Figure** **5**). The wear surface of the cobalt alloy presented in Figure 5a is different from the titanium alloy wear surface shown in Figure 2a. We hypothesize that the fine and coarse grooves along the wear track of the cobalt alloy were produced primarily due to microploughing by the wear debris and the raised shelves, respectively, formed on the titanium surface. Since not all the debris particles or shelves must have been in direct contact with the cobalt alloy surface, we refrain from directly comparing the size of the grooves to the sizes of the features on the titanium alloy surface.

#### 2.3.2 Subsurface of the Cobalt Alloy

A STEM‐BF image of the subsurface of a groove on the cobalt alloy is given in Figure 5b. The immediate subsurface shows crystalline features representing stacking faults or twins. The higher density of defect structures in the upper layer could be attributed to plastic deformation due to the mechanical contact. Correlative STEM BF‐APT reconstruction of the cobalt alloy (performed on a specimen lifted from the region marked in Figure 5b) is given in Figure 5c,d, respectively. No grain refinement leading to nanocrystallinity on a scale comparable to the titanium alloy subsurface (which was rendered partially amorphous) is detected. Instead, the features of the cobalt alloy subsurface resemble the initial microstructure of the cobalt alloy (Figure 1e).

#### 2.3.3 Chemical Composition of the Subsurface of the Cobalt Alloy

In order to preserve surface features during sample preparation, the correlative STEM‐APT specimen was covered by a platinum protective layer. Excluding the upper platinum layer on the APT specimen, the 1D composition profile of the subsurface (Figure 5e) appears to be similar to the initial homogenous composition of the cobalt alloy (Figure 1f). Unlike the titanium alloy subsurface, no external ions (such as oxygen or chlorine) are present. The platinum rich layer contains 0.5 at% of CrO ions and ≈5% Mo. Although the exact reason why the platinum rich region is enriched by molybdenum is unknown, the presence of CrO indicates a chromium rich thin passivation layer.

### 2.4 Composition of the Tribomaterial and Serum after the Fretting Tests

Tribomaterial is formed during fretting due to wear particles that are not ejected from the system because they agglomerate and mix with denatured proteins (proteins are present in the bovine calf serum).^52^, ^53^, ^54^ This interaction results in formation of a metallic‐oxidic‐organic tribomaterial that remains attached to the surfaces.^55^ The metal content of the tribomaterial containing soap solution after cleaning the surfaces as well as the serum in which the fretting surfaces were immersed during the test is presented in **Table** **1**.

The higher amount of titanium detected in the tribomaterial compared to the serum indicates that titanium wear particles generated during the fretting remain in the tribomaterial and are not transported into the serum. The higher chromium content compared to cobalt in the tribomaterial (chromium/cobalt concentration ratio ≈ 2.9) indicates the presence of chromium oxides. In contrast, the chromium and cobalt concentration in the serum is at a ratio of 0.28, closer to that of the cobalt alloy composition of 0.41, suggesting that corrosion of the cobalt alloy takes place releasing cobalt, chromium and molybdenum into the serum.

**Table 1:** Composition of the tribomaterial containing soap solution and serum after the fretting wear tests as measured by ICP‐OES

	Co [µg kg^−1^]	Cr [µg kg^−1^]	Mo [µg kg^−1^]	Ti [µg kg^−1^]
Tribomaterial	22 ± 5	64 ± 8	11 ± 4	896 ± 31
Serum	680 ± 57	194 ± 20	44 ± 1	<40

## 3 Discussion

The goal of this study was to pinpoint the origin of metal ion release from titanium–cobalt alloy taper junctions in hip implants during fretting in a proteinaceous environment as well as reveal how the titanium alloy is able to wear the harder cobalt alloy. Since wear and corrosion phenomena take place simultaneously in this tribosystem, we realized that it is necessary to probe not only the wear surfaces, but also deploy a multiscale probing approach capable of identifying both chemical and structural changes in the subsurface of the material.

**Figure 6:**
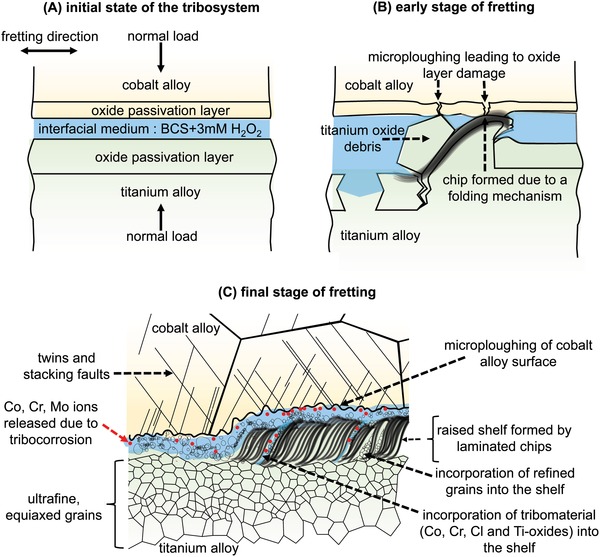
Schematic representation of the mechanism of fretting corrosion and metal ion release in the titanium–cobalt alloy couple. A) initial state of the tribosystem, with the loading and fretting directions marked. B) early stage where the titanium oxide wear debris ploughs the titanium alloy surface to form a chip that remains on the surface due to a folding mechanism. The debris and chips formed on the titanium alloy surface can microplough the cobalt alloy surface, thus damaging the passivation layer of the cobalt alloy. C) final stage of the fretting test where the chips formed on the titanium alloy surface pile up on each other during successive fretting strokes and are laminated together to form raised shelves. The shelves contribute to microploughing of the cobalt alloy surface. When the passivation oxide layer of the cobalt alloy is compromised, dissolution of cobalt, chromium, and molybdenum into the interfacial medium takes place. The metal ions from the corroding cobalt alloy, the oxide‐particle laden liquid fraction of the tribomaterial formed between the surfaces as well as refined grains from the titanium alloy are all incorporated into the shelf during the folding process.

After the fretting tests, the cobalt alloy has shallow grooves on the surface and slight plastic deformation in the subsurface region. The titanium alloy surface has raised shelves, separated by troughs filled with grainy debris. The subsurface of the shelves is comprised of fragmented α‐crystals, oxide bands, the refined matrix and intermixed zones, all laminated together. Our observations are consistent with one of the few subsurface studies on a Co–Cr–Mo alloy neck/Ti‐12Mo‐6Zr‐2Fe alloy stem couple from an implant retrieval where grain refinement and oxides in the titanium subsurface were observed alongside loss of nanocrystallinity on the cobalt alloy side.^41^ Based on our findings, we propose the failure sequence during fretting of the cobalt–titanium alloy couple, under gross slip and boundary lubrication conditions, that leads to wear and tribocorrosion (schematically illustrated in **Figure** **6**).

### 3.1 Initial State of the Cobalt Alloy–Titanium Alloy Tribosystem

The initial state of the tribosystem prior to fretting has been schematically illustrated in Figure 6a. The Co–Cr–Mo has higher oxide fracture and interfacial adhesion strengths than Ti‐6Al‐4V.^39^ The nanoindentation hardness of chromium oxide is 29.1 GPa, while that of TiO is 19.6 GPa and TiO_2_ is 6–10 GPa.^56^ Hence, the abrasive wear of the titanium alloy by the cobalt alloy can generate titanium wear debris, which has been found to primarily consist of titanium oxides.^44^

### 3.2 Early Stage of Wear in the Cobalt Alloy–Titanium Alloy Tribosystem—Formation of Chips on the Titanium Alloy Surface

When abraded by the titanium oxide debris, we hypothesize that the material of the titanium alloy is shifted in front of the groove, generating chips on the surface of the titanium alloy similar those observed in the case of microcutting (Figure 6b).^57^

### 3.3 Intermediate Stage of Wear in the Cobalt Alloy–Titanium Alloy Tribosystem: Formation of Shelves on the Titanium Alloy Surface

However, these chips are not torn‐off and emitted as wear particles but instead pile up on each other. On successive fretting strokes, this pile‐up of chips lifts the subsurface to form the raised shelves found on the titanium alloy wear surface (Figure 6c). This hypothesis is based on the presence of the laminated subsurface beneath the shelves (Figure 2b), which contains severe plastic deformation resembling primary shear bands. The pile‐up of chips could occur via a folding mechanism, as was observed in the case of an unconstrained plastic flow in pure copper.^58^, ^59^ The delamination of chips is prevented by the presence of polycrystalline grains via a bulging and coalescence mechanism,^59^ which effectively folds the primary shear band to form the chip (Figure 6b). The equiaxed nanograins formed in the titanium alloy (zone 2 in Figure 2b) could also facilitate this folding mechanism in the region above it (zone 1 in Figure 2b).^59^ During the folding process, surface oxides (Figure 4a,b) and part of the tribomaterial containing oxides enriched in chromium and cobalt (from the cobalt alloy counterbody), as well as chlorine (from the bovine calf serum) are incorporated in the shelf (Figure 4c). The intermixing of matrix phases with external constituents in the shelves creates a partially amorphous composite structure enriched by oxygen. This process appears similar to mechanical milling, which would generate a new alloy in the presence of oxygen,^60^ as well as mechanical mixing, that would generate a new metal‐oxide composite.^55^ Although shelves with adjacent delaminations on the wear track would normally be attributed to the wear mechanism of surface fatigue,^61^ we exclude this possibility due to the absence of cracks below the surface, that would form in such a case due to cyclic contact stresses under predominantly elastic interaction of the bodies in contact.^62^, ^63^, ^64^

### 3.4 Cobalt Alloy–Titanium Alloy Tribosystem after the Concurrent Occurrence of Wear and Corrosion

The thus generated shelves on the titanium alloy surface as well as the grainy debris microplough the cobalt alloy surface, as is evident from the grooved features on the cobalt alloy wear surface (Figure 5a). The detection of chromium and titanium oxides in the titanium alloy subsurface (Figure 4c) is clear evidence that some part of the chromium‐rich passivation layer of the cobalt alloy as well as titanium‐rich oxide passivation layer of the titanium alloy is removed during the fretting. Compromising the integrity of the oxide passivation layer leads to corrosion of the alloys and dissolution of the constituent elements into the surrounding medium until repassivation takes place.^37^, ^39^ From thermodynamic data,^55^, ^65^ the cobalt alloy is expected to undergo active dissolution of cobalt, transpassive dissolution of molybdenum, and passivation by chromium and the titanium alloy is expected to undergo passivation by titanium, passivation by aluminium, and dissolution of vanadium under the conditions of the fretting tests. The presence of reactive oxidative species (such as H_2_O_2_ which was added to the interfacial medium), which are present in highly inflamed synovial fluid, are known to increase the corrosion potential of the cobalt alloy making it more susceptible to corrosion.^66^ The faster regeneration of the surface oxide in the titanium alloy compared to the cobalt alloy^67^, ^68^ could be the reason why little titanium was detected in the serum that was enriched in cobalt, chromium, and molybdenum instead (Table 1). In addition, the limited plastic deformation seen in the subsurface of the cobalt alloy after the fretting tests (Figure 5b) could also be attributed to the higher rate of tribocorrosion that takes place in the cobalt alloy. This tribocorrosion supersedes any change in the substructure of the cobalt alloy due to deformation. Since each subsequent fretting stroke takes place on a “fresh” surface, the structure and composition of the cobalt alloy subsurface after the fretting test resembles the initial microstructure before the fretting tests. In addition, the absence of a passive film can cause dislocations (generated by the shear stress at the interface during fretting) to be attracted to the bare metal surface and thus be annihilated.^69^ Similarly, the presence of a passive film leads to a higher dislocation density in the bulk of the material, leading to grain refinement down to nanograin formation as well as accelerating mechanical wear, although the quantitative fractions of the corrosive and the mechanical part are unknown.^70^ This is also a plausible reason for how the slower repassivation kinetics of the cobalt alloy leads to a lack of nanocrystallinity in the subsurface of the cobalt alloy compared to the titanium alloy. The final state of the tribosystem is schematically depicted in Figure 6c.

Our observation of the incorporation of (formerly surface) titanium and chromium oxides in the subsurface of the titanium alloy is consistent with the recent discovery of Cr_2_O_3_ and amorphous TiO_2_ in the tissues surrounding corroded dual taper Co–Cr–Mo/Ti‐12Mo‐6Zr‐2Fe junctions^24^ (This implant design has been recalled from the market due high early failure rates). The correlation between the titanium and chromium oxides in periprosthetic tissues suggest that the same oxides that we observed being folded into the titanium alloy could also be expelled from the gap (possibly after longer fretting tests or different fretting regimes) and accumulate in surrounding tissues, leading to ALTRs. It is also to be noted that proteins present in the bovine calf serum could influence the corrosion behavior of the alloys.^71^ Increasing protein concentration has been found to decrease the oxide film resistance of Co–Cr–Mo alloys.^72^ Albumin can limit the adsorption of phosphate ions on Co–Cr–Mo alloys, thus accelerating corrosion, but can also modify the passive film properties and, by acting as a cathodic inhibitor, reduce the corrosion rate.^73^ The role of the proteins present in bovine calf serum on the dissolution of the alloys could be investigated by analyzing the organic components of the tribofilm formed between the alloy surfaces and will be the subject of our future work.

## 4 Conclusion

Although implant failure in the body is a complex, multifactorial process, the chosen contact conditions and interfacial medium in these in vitro tests have allowed us to evaluate the origin of metal ion release during fretting corrosion in implants with cobalt‐titanium alloy taper junctions at the near‐atomic scale. A folding mechanism taking place during the fretting generates raised shelves on the titanium surface. These shelves on the titanium alloy microplough the cobalt alloy and hence promote tribocorrosion in the cobalt alloy. As a result, the constituent elements of the cobalt alloy can be found on the titanium subsurface and metal ions are released into the surrounding fluid. This conclusion provides a mechanistic explanation why Ti‐6Al‐4V and Co‐29Cr‐6Mo should not be paired in hip implant taper junctions in the presence of a corrosive medium when micromotion between both materials cannot be ruled out. Better understanding of this failure sequence is relevant to develop new titanium alloys for the taper junction that do not form shelves during fretting or explore alternative alloys that are less susceptible to tribocorrosion under conditions where the taper junction is not fully locked.

## 5 Experimental Section

##### Fretting Wear Tests

A custom built testing rig attached with a potentiostat was used for this study. A cobalt (Co‐29Cr‐6Mo) alloy pin, mechanically polished to a near‐flat convex surface (radius = 8–10 m) and a titanium (Ti‐6Al‐4V) alloy cylinder of diameter = 13 mm were used. The titanium cylinder was held by a sample adapter such that the long axis stays vertical and the polished surfaces of two cobalt alloy pins (clamped horizontally) touched the curved surface of the titanium alloy cylinder from opposite sides, as schematically illustrated in Figure 1G. This type of placement of the cylinder and pins created a more realistic condition, similar to the placement of head‐neck assembly of the hip implants in the body, where gravity might play a role in redistributing the wear particles and tribomaterial. The whole setup was immersed in 10 mL bovine calf serum (BCS) with 3 mm H_2_O_2_, at a pH value = 7.6 (average pH in the body) and held at a constant temperature of 37 °C (average body temperature). H_2_O_2_ has been proposed to be among the reactive oxidizing species generated by cells when there is a strong inflammatory reaction or infection and has been known to increase the corrosion susceptibility of both cobalt and titanium alloys.^74^ Although H_2_O_2_ has been mostly used in corrosion studies,^75^, ^76^ it has been used in a few wear studies as well.^77^, ^78^ A force of ≈18.5 ± 4 N was applied normal to the curved surface of the titanium cylinder through the cobalt pins. With the geometry of the polished surface of the cobalt alloy, the maximum Hertzian pressure on the cylinder by the normal forces was calculated to be ≈0.17 GPa. At this state, the micromotion between the counterbodies was simulated by oscillating the titanium cylinder vertically with an amplitude of displacement = 50 µm and a frequency of oscillation = 4 Hz. 50 000 cycles of oscillations were performed. The forces and displacements were chosen such that the fretting remains in a gross slip regime by confirming that the work criterion was greater than 0.2.^79^ Each fretting experiment was replicated three times. Thus, six samples (two per Ti‐6Al‐4V rod) were subjected to 50 000 cycles of fretting. On analysis of the wear tracks, all six samples showed the elevated shelf‐like features described in the manuscript. It is to be noted that elevated shelves were also found in the wear tracks of titanium alloy–cobalt alloy pairs fretted in pure BCS as extensively characterized by SEM in ref. 44. The composition of the serum after the fretting test was analyzed using inductively coupled plasma‐optical emission spectroscopy (ICP‐OES). The wear surface was then sonicated in enzymatic soap and the composition of the resultant solution, which was assumed to be the composition of the tribomaterial formed between the surfaces, was analyzed by ICP‐OES as well.

##### Correlative TEM/APT Analysis

A protocol developed at MPIE, Düsseldorf was implemented for correlative TEM/APT analysis.^45^ For this purpose, custom‐made specimen holders compatible for both a 200 kV JEOL 2200FS TEM (equipped with STEM‐HAADF and NBD instrumentation) and a CAMECA LEAP 5000XR atom probe microscope were used for the data acquisition. While the TEM provides nanoscale structural and crystallographic information, the APT gives quantitative 3D compositional information with near atomic resolution and detection sensitivity of 10 ppm for all elements. After the TEM analysis, the specimen was cleaned using focused ion beam milling at 5 kV and 15pA, resulting in the removal of an approximately 10 nm thick layer of material from the outer periphery of the sample. APT acquisition was carried out in laser pulse mode, a pulsing of 200 kHz with an energy of 40 pJ at specimen temperature ≈50 K was used for APT data acquisition. The correlative samples and TEM overview lamellae were prepared using a dual beam focused ion beam instrument with a Ga source.

## Conflict of Interest

The authors declare no conflict of interest.
